# A rupioid rash

**DOI:** 10.1093/rheumatology/kead073

**Published:** 2023-02-08

**Authors:** Nikolaos Fytanidis, Charalampos Papagoras

**Affiliations:** First Department of Internal Medicine, Democritus University of Thrace, University Hospital of Alexandroupolis, Alexandroupolis, Greece; First Department of Internal Medicine, Democritus University of Thrace, University Hospital of Alexandroupolis, Alexandroupolis, Greece

A 72-year-old man presented for a thick hyperkeratotic skin rash on the shins that had erupted 7 months earlier after minimal local trauma (Fig. 1A, B). Topical treatments with glucocorticoids and antibiotics were ineffective, although a slight improvement was noted during summer. Additionally, low-grade fevers and an asymmetric oligoarthritis of the upper and lower limbs and dactylitis of the thumb and fourth toe on the left appeared over the last weeks. Mild non-specific onychodystrophy of the toes was found, whereas no inflammatory back pain or enthesitis was present. Plain radiographs did not reveal disease-specific deformities, erosions or osteo-proliferative lesions. Laboratory tests showed raised inflammatory markers (CRP: 17.7 mg/dl, ESR: 107 mm/h), leukopoenia (2.580/μL) and anaemia (haemoglobin: 10.3 g/dl). Differential diagnosis of the rash included crusted scabies, secondary syphilis, neutrophilic dermatoses, histoplasmosis and a hyperkeratotic psoriasis variant [1]. Skin biopsy revealed psoriasiform acanthosis, thick parakeratosis, dense neutrophilic infiltration and microabscess formation (Fig. 1C). Based on the type of musculoskeletal involvement and the skin histopathology, the patient was diagnosed with psoriatic arthritis with rupioid psoriasis. Treatment with medium-to-low dose systemic glucocorticoids and infliximab led to arthritis remission, skin (Fig. 1D) and laboratory improvement. *Rupioid* psoriasis (derived from the Greek word ρύπος = dirt) is a rare hyperkeratotic variant of psoriasis characterized by well-demarcated plaques with thick, dirty-appearing adherent crusts. It is associated with local treatment failure [2], psoriatic arthritis and male sex and it may present challenges in the diagnosis of patients with arthritis and systemic inflammation. Written informed consent has been obtained from the patient.

**Figure 1.**
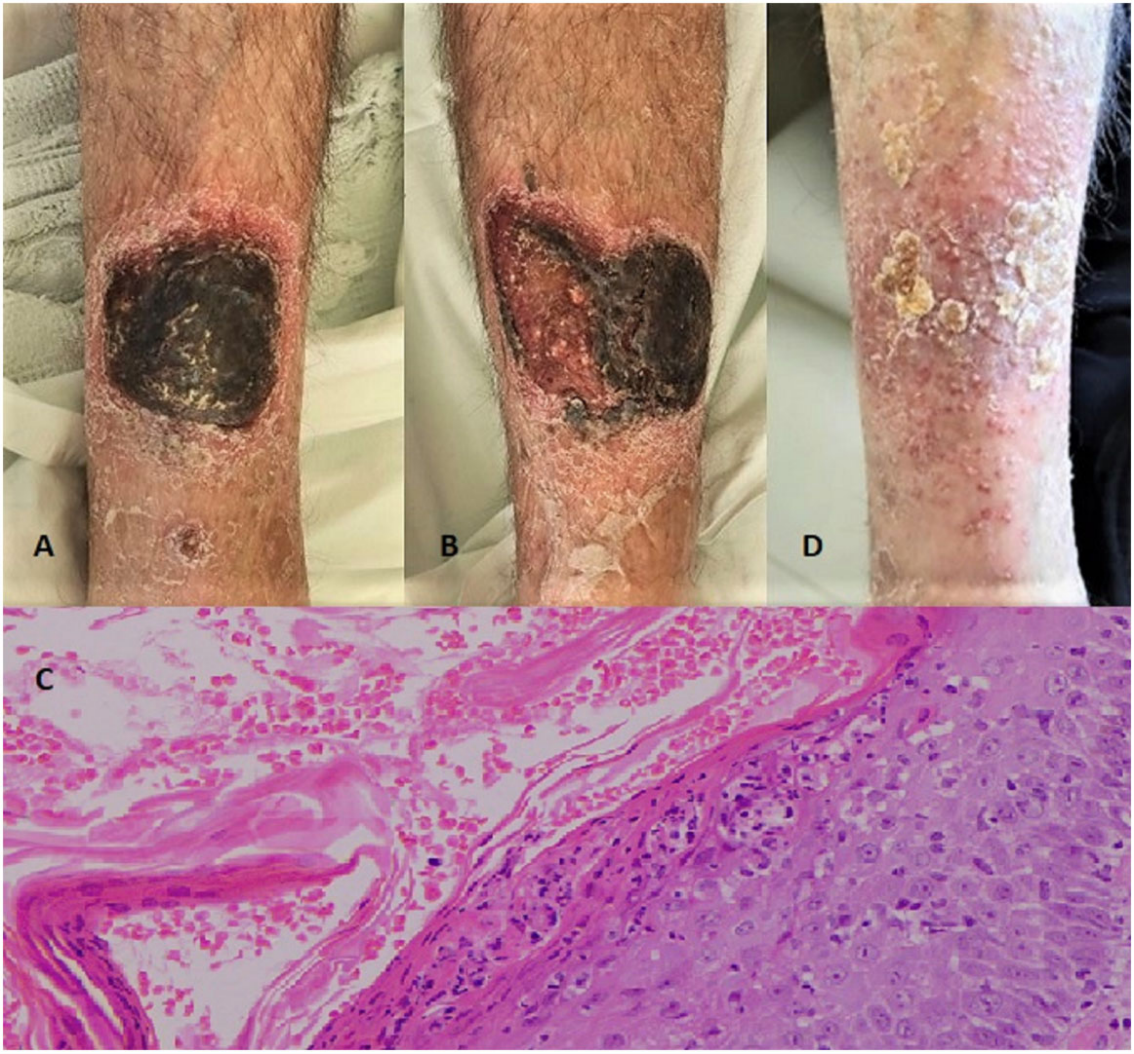
Rupioid psoriasis: clinical and histological appearance. Thick hyperkeratotic plaques on the shins (**A**, **B**) and the corresponding histology image (×20) showing psoriasiform acanthosis, parakeratosis and neutrophilic infiltrates with microabscess formation (**C**). Clinical improvement after treatment with infliximab and glucocorticoids (**D**)

## Data Availability

The data will be shared on reasonable request to the corresponding author.
